# On the limits of the relation of disgust to judgments of immorality

**DOI:** 10.3389/fpsyg.2015.00951

**Published:** 2015-07-15

**Authors:** Mary H. Kayyal, Joseph Pochedly, Alyssa McCarthy, James A. Russell

**Affiliations:** Department of Psychology, Boston College, Chestnut HillMA, USA

**Keywords:** emotion, morality, anger, disgust, language

## Abstract

Two correlational studies (*n*s = 400; 90) examined the association of judgments of immorality and disgust (hypothesized in much current research and theory). Across 40 scenarios in Study 1, immorality was positively correlated with negative emotions, especially anger. With anger partialed, disgust was significantly, but weakly, correlated with immorality, *r*(38) = 0.22, *p <* 0.05. Study 2 asked whether the immorality-disgust correlation is due to a confound: immoral events often include elements implicitly or explicitly implying pathogens, such as blood or semen. Across 22 scenarios, those implying pathogens were associated with disgust, but those without pathogens, whether moral or immoral, rarely were. We propose that the relation between disgust and immorality is largely coincidental, resulting from (a) using the word *disgust* to express anger with or even dislike of immoral acts and (b) the presence of incidental elements capable of eliciting disgust.

## Introduction

Most of us find some events both immoral and disgusting (for example, sex with a rotting corpse). The association of immorality with disgust in such events might be simply a coincidence, just as we find some immoral events occur on a Tuesday. Proclaiming an immoral event as disgusting might also be a metaphorical way of speaking. That is, most of us feel bad about immoral events – they anger us, we dislike them – and we might express those feelings by exclaiming, “that’s disgusting!” just as we might say, “that stinks!” Doing so might serve to induce stronger feelings in the listener.

On the other hand, much theorizing and research in moral psychology today suggests that the association of immorality with disgust is more than coincidence or metaphor. These accounts are part of a broader enterprise in which emotion more generally, rather than just cold reasoning, is involved in moral judgment (Kagan, 1984; Shweder and Haidt, 1993; Prinz, 2006). Avramova and Inbar (2012, p. 170) observed that, “most current psychological accounts of moral judgment hold that affect plays an important role.” And, disgust is a prime focus in these accounts. Chapman and Anderson (2014, p. 341) proclaimed an “emerging consensus that disgust plays a role in human morality.” Indeed, some theorists posit a role of disgust for the entire domain of moral judgment (Wheatley and Haidt, 2005; Schnall et al., 2008; Chapman et al., 2009; Chapman and Anderson, 2013, 2014). An evolutionary account of disgust has been offered in which disgust began as a mechanism to avoid disease carried by food, but was co-opted to avoid other potential harms, including social disruption and violation of moral norms (Haidt et al., 1994; Tybur et al., 2009). Darley and Pittman (2003) proposed that “moral outrage” underlies retribution, with moral outrage including disgust, anger, and contempt. On one theory, some events are found immoral, at least in part, *because* they are disgusting: Haidt (2001) theorized that moral judgments are often *post hoc* rationalizations of gut-level intuitions, often disgust reactions. Russell and Giner-Sorolla (2012, p. 1) not only agreed on disgust’s causal role on moral judgments – calling disgust an “irrational and inflexible influence on our moral judgments” – but also suggested that disgust therefore plays “a powerful role in shaping cultural attitudes, politics, and law.” Focus on the role of disgust in moral judgment has reached beyond psychology. Kelly’s (2014) philosophical account of moral judgment adopted the evolutionary co-optation account in which disgust now serves the function of avoiding socio-moral violations.

Other theorists, however, contend that the role of disgust is limited to certain subclasses of immorality such as violations of purity and autonomy (Rozin et al., 2009; Preston and Ritter, 2012; Graham et al., 2013; Feinberg et al., 2014). We return to the claim that the role of disgust is limited to only certain classes of immoral events after considering the broader claim that disgust is associated with judgments of immorality in general (Chapman and Anderson, 2014). Although Avramova and Inbar (2012, p. 175) concluded that there is “ample support” for at least a weak association between disgust and judgments of immorality, we were less convinced by available evidence. Consider the evidence taken to support an association between immorality and disgust. When people freely list the things that disgust them, the list is long and varied, but includes some events that are also judged immoral: tabooed sexual acts, brutal beatings, cheating, stealing, lying, and hypocrisy (Haidt et al., 1994). But, as we said, some events are both disgusting and immoral, but that fact does not establish an association between disgust and immorality beyond coincidence (Inbar and Pizarro, 2014), nor does it deny an association between morality and any other negative emotion (such as anger). Words such as *disgust* are not used in everyday speech as if they were precise scientific terms, but rather in a loose and open-ended manner (Nabi, 2002). When asked to judge how *disgusted* various immoral events make them feel, people rate some immoral events as disgusting (Tybur et al., 2009). Again, such events might be both immoral and disgusting by coincidence, but an additional problem sometimes arises: participants are sometimes limited to one emotion option—*disgust*. Given that constraint, participants might have been especially inclined to use the word *disgust* to express their general dislike. Our criticism is consistent with the interpretation that the word *disgust* is being used metaphorically (Royzman and Sabini, 2001; Bloom, 2004; Oaten et al., 2009).

Some studies offered evidence that immoral events are associated with moral disgust more than with other emotions. For example, participants say that they feel moral disgust more intensely than any other emotion (when given a list including anger, contempt, sadness, and fear/anxiety) in response to a wide range of immoral events (Hutcherson and Gross, 2011). But again, a methodological point arises: participants responded to the label *moral disgust* rather than just *disgust*, and the word *moral* did not precede any of the other emotion options. Affixing the word *moral* to an emotion word increases that emotion’s moral relevance (Russell et al., 2013). Thus, the association between moral disgust and immoral events may have been due to the word *moral*. Russell et al. (2013) found that, when this problem is corrected, anger is indeed the more prominent response to immorality.

Some studies have offered evidence that immoral events are associated with disgust – rather than moral disgust – specifically. In these studies, however, disgust was associated with a subset of immoral events, primarily those that included (explicitly or implicitly) possible contact with pathogens through sex, blood, rotten food, a corpse, and the like (Rozin et al., 1999; Russell et al., 2013). Disgust was less frequently associated with immoral events that lacked contact with matters not themselves disgusting—events such as theft or disrespect. As pointed out in a recent review (Royzman et al., 2014), the studies that most clearly support an association between disgust and immorality confound immorality with elements that are disgusting anyway because of an association with pathogens.

Some studies have attempted to rule out the pathogen confound by showing, for example, that participants report feeling disgusted by immoral events that do not explicitly involve pathogens such as sex, violence, and death. In a study directly examining the pathogen confound, participants reported feeling disgusted in response to knowingly eating cloned meat explicitly said to be disease-free (Gutierrez et al., 2012). Again, we are less convinced by such evidence. Humans and other animals cannot detect pathogens directly, but must rely on detectable correlates, especially the vectors and visible consequences of pathogens. Sex, violence, and death are just such correlates and might be evolutionarily based signs of possible pathogens. That is, during phylogenesis and ontogenesis, disgust could not be associated with disease-causing pathogens directly (which are beyond our ability to perceive), but only with possible correlates of disease. Eating something never eaten before risks exposure to pathogens. A verbal statement that something is disease-free might be insufficient to bypass the association of disgust with meat not previously eaten.

Here we report two studies that explore the hypothesis that judgments of immorality are tied to disgust reactions so weakly that the association might be coincidence or a metaphorical way of speaking. The first study is on the role of disgust in moral judgments generally, the second on the possibility that those events found both disgusting and immoral contain, at least implicitly, signs of pathogens.

## Study 1: Anger, Not Disgust, is Strongly Related to Moral Judgments

To test the hypothesis that disgust is not the dominant emotional reaction to actions judged immoral, Study 1 examined the correlation between judgments of morality and emotion. We created 40 scenarios in which a protagonist carried out some action. In a between-subject design, each participant read one scenario and rated (a) the degree to which he or she felt each of five emotions: happy, disgusted, angry, sad, and scared, and (b) how morally bad or morally good he or she found the action of the protagonist. We created different scenarios such that roughly half of the scenarios would be judged morally good and half morally bad. Our anticipations as to morality and as to emotions, however, played no role in the data analysis. We simply examined the correlations between morality and emotion ratings.

### Method

#### Participants

An online sample of 400 participants (*n* = 186 males; 18–67 years, *M*_age_ = 32 years) was recruited using Amazon Mturk. Participants gave written informed consent, and the study was approved by the ethical committee of Boston College.

#### Scenarios

The 40 scenarios are given in Appendix A.

#### Survey Ratings

Each scenario was rated by 10 participants. Participants were randomly assigned to receive one of the 40 scenarios, with the proviso that the total number receiving each was 10. Participants were instructed, “Imagine hearing about or witnessing the following scenario. In the story, one character, Casey (indicated in bold), is the focus of the two questions below.” Participants made an emotion judgment and, separately, a morality judgment.

#### Emotion Judgment

After reading the scenario, the participant was asked, “How would hear about or witnessing this scenario make you feel?” The participant rated the degree to which they felt each of five emotions: (disgusted, angry, sad, scared, and happy) on a scale ranging from 0 (not at all) to 7 (extremely). The instructions explicitly stated that they could select as many or as few emotions as they wanted.

#### Morality Judgment

After the emotion judgment, the participant was asked to judge “how morally bad or morally good” the protagonist’s action was by rating it as extremely morally bad, moderately morally bad, barely morally bad, barely morally good, moderately morally good, or extremely morally good, coded as -3, -2, -1, 1, 2, and 3, respectively.

### Results

#### Moral Judgment

Participants largely agreed with our a priori categorization of the scenarios as immoral or moral. The mean morality judgment (on the -3 to +3 scale, with no 0 option) for the 20 scenarios anticipated to be immoral was -2.00; and for the 20 scenarios anticipated to be moral it was 1.90, *t*(398) = 29.15, *p* < 0.001. Dichotomous scores showed the same pattern. Ninety-three percent of the scenarios anticipated to be immoral were indeed categorized as immoral (i.e., received a score of -1, -2, or -3); 88% of scenarios anticipated to be moral were indeed categorized as moral (i.e., received a score of 1, 2, or 3).

#### Emotion Judgment

Most scenarios were associated with several emotions, albeit to different degrees. Different scenarios were even modally associated with different emotions – disgust, anger, sadness, fear, and happiness – in roughly equal proportion. Appendix A gives the mean intensity of each emotion for each scenario.

#### Relation between Moral Judgments and Emotion Judgments

**Table 1** shows that the zero-order Pearson correlation between emotion intensity and moral judgment was negative and significant (all *p*s < 0.01) for each of the negative emotions. Thus, the more disgusting, angering, saddening, or frightful a story was judged to be the more immoral it was also judged to be. For happiness ratings, the corresponding correlation failed to reach significance. (Similar results were obtained when emotion ratings and moral judgments were dichotomized; phi correlations are available upon request. Similar correlations were obtained when Spearman rank correlations were calculated. There is some controversy concerning whether data from Likert response formats should be analyzed with parametric or non-parametric procedures; for a review see Carifio and Perla, 2007).

**Table 1:** Correlations (*N* = 400) between emotion intensity and morality, Study 1.

		Partial correlation	
	Correlation	Controlling for anger	Controlling for disgust
Anger	-0.53^∗∗^	–	-0.36^∗∗^
Disgust	-0.46^∗∗^	-0.22^∗^	–
Fear	-0.15^∗∗^	-0.06	0.11^∗^
Sadness	-0.14^∗∗^	-0.02	-0.13^∗∗^
Happiness	0.07	0.04	0.04

*^∗∗^denotes significance at 0.01 level; ^∗^denotes significance at 0.05 level.*

Emotion judgments were also correlated with each other, and so the question is which emotions correlated significantly with morality judgments when the others are controlled. Morality judgments were more strongly related to anger than to disgust, as indicated by partial correlations (*N* = 400) between disgust and morality when controlling for anger and, separately, between anger and morality when controlling for disgust, as is shown in **Table 1**. The correlation between disgust and morality was reduced when controlling for anger.

A stepwise multiple regression analysis explored which emotion judgments significantly predicted moral judgments. The five predictors were participants’ intensity rating for each emotion: disgust, anger, sadness, fear, and happiness; the dependent variable was the moral judgment intensity score. At step 1, anger explained a significant proportion of variance in morality judgments, *R*^2^ = 0.29, *F*(1,398) = 158.48, *p* < 0.001; for anger, β = -0.56, *t*(396) = 12.59, *p* < 0.001. At step 2, both anger and disgust judgments entered the equation which resulted in an incremental change of 0.03 in *R*^2^, *F*(1,397) = 19.77, *p* < 0.001, and explained a significant proportion of variance in moral judgments, *R*^2^ = 0.32, *F*(2,397) = 92.86, *p* < 0.001; both anger and disgust were significant predictors, for anger, β = -0.39, *t*(396) = 7.76, *p* < 0.001; for disgust β = -0.22, *t*(396) = 4.45, *p* < 0.001. Judgments of sadness, happiness, and fear failed to add a significant increment in predicting morality judgments. Because of multicollinearity, an alternative interpretation of these results is that negative affect predicts morality judgments.

### Conclusion

Morality judgments were significantly correlated with each of the negative emotions included, rather than to one specific emotion. In general, events that were judged as more emotionally bad (those that make us feel disgusted, angry, sad, or scared) were also judged as more morally bad. However, events that were judged as emotionally good (those that made respondents feel happy) were not also judged as more morally good.

Anger and disgust were more strongly associated with moral judgments than were the other negative emotions. Our findings with anger resonate with Darley and Pittman’s (2003) focus on moral outrage, with Rozin et al.’s (1999) inclusion of anger as part of the reaction to immorality, with Russell et al.’s (2013) evidence that anger is the more powerful predictor of judgments of immorality, and with Royzman et al.’s (2014) similar conclusion. Okimoto and Brescoll (2010, p. 933) assessed “moral outrage” with items for anger, contempt, and disgust. Factor analysis indicated that “participants did not distinguish among” them.

When anger was controlled, the association between morality and fear, and morality and sadness, was negligible. Anger rather than disgust was most strongly related to moral judgments. And, only anger remained significantly associated with moral judgments when controlling for disgust. In contrast, disgust lost much (although not all) of its association with moral judgments when controlling for anger.

## Study 2: Why Might Disgust and Immorality be Associated?

Study 1 found a negative correlation (albeit weak) between disgust and immorality when anger was statistically controlled, and the question is why. One possibility is that immoral events elicit disgust. Another possibility is that feeling disgust makes people judge events as immoral. Another is that judging an event as immoral is expressed by calling it disgusting, a way of expressing or inducing intense dislike or anger. Yet another possibility is that immoral events tend to include disgusting elements: violence can include blood; tabooed sex can imply the possibility of sexually transmitted diseases, and so on. Indeed, scenarios for Study 1 with the highest judgments of disgust involved sex, food, and saliva.

Study 2 examined the possibility advanced by Royzman et al. (2014) that disgust co-occurs with judgments of immorality when the immoral event includes signs of potential exposure to pathogens, but not otherwise. If so, the relation between disgust and moral judgments is largely coincidental, based on confounding immoral events with elements capable of eliciting disgust. Study 2 was also correlational. We created 22 scenarios in a roughly 2 × 2 design by crossing “immoral”–“moral” with “with pathogens”–“without pathogens.” That is, each scenario was hypothesized to depict either an immoral (12 scenarios) or not immoral (10 scenarios) event that either included (implicitly or explicitly) potential pathogens (13 scenarios) or lacked potential pathogens (9 scenarios). By “pathogen,” we mean possible sources of infection, including sexual acts and body fluids or products (such as saliva, blood, vomit, feces; Tybur et al., 2009). We also varied our method, participant population, and rating task slightly from that in Study 1 to ensure that our general results were robust across such minor variations. For each scenario, participants selected the single best word to describe their emotional reaction (from a list of 9 chosen to provide all the basic emotions plus outrage and contempt as possible expressions of moral dislike) to the event depicted in the scenario.

### Method

#### Participants

Ninety Boston College undergraduate students (18–21 years, 30 male, 55 female, five unspecified) completed the online survey in return for course credit. Participants gave written informed consent, and the study was approved by the ethical committee of Boston College.

#### Scenarios

We created 22 scenarios in a 2 × 2 design, crossing the moral and pathogen content. Appendix B gives the scenarios in these four categories: Immoral with Pathogens, Immoral without Pathogens, Moral with Pathogens, and Moral without Pathogens. The scenarios were presented to participants in random order.

#### Survey Sections

There were two sections presented in counterbalanced order. In each section, participants were instructed to respond specifically to the actions of the protagonist or antagonist in the story (bolded in Appendix B).

#### Emotion Judgment

For each scenario, participants were asked to describe how they felt about the event described in the scenario by selecting one emotion from a list of nine labels: *happy, sad, angry, scared, surprised, contemptuous, disgusted, outraged*, or *none of the above.* The participant then rated the intensity of the selected emotion on a scale ranging from 1 (*barely*) to 7 (*extremely*). (In the analyses, a score of 0 was given when the participant did not select the emotion.) The *none of the above* option required any intensity rating. Participants were asked to respond to each scenario independently of the others so that an emotion label could be selected as many times as appropriate across scenarios.

#### Morality Judgment

For each scenario, participants rated the extent to which the event described was immoral on a scale ranging from 0 (*not immoral at all*) to 6 (*extremely immoral*).

### Results

#### Manipulation Checks

For each scenario, Appendix B gives disgust and immorality scores. Intensity scores are means; % yes is the percentage of participants who selected a value other than zero.

#### Immorality

Participants agreed with our a priori categorization of the scenarios as ‘immoral’ or ‘moral.’ The mean immorality intensity score (on a 0 to 6 scale) for the 12 Immoral scenarios was 4.5; the corresponding figure for the 10 Moral scenarios was 0.3, *t*(89) = 6.72, *p* < 0.001. Each immoral scenario was rated as significantly more immoral than any moral scenario. The dichotomous scores yielded similar results. A majority rated all 12 immoral scenarios as immoral, and all 10 moral scenarios as moral. The mean percentage of *yes* on the immorality question for the 12 immoral scenarios was 99%; the corresponding figure for the 10 moral scenarios was 4%.

#### Pathogens

Participants also agreed with our *a priori* assumption that scenarios ‘with pathogens’ were more disgusting than those ‘without pathogens,’ although agreement was moderate. Scenarios with pathogens were associated with disgust more intensely than scenarios without pathogens (mean disgust intensity = 1.9 versus 0.2, respectively), *t*(89) = 22.76, *p* < 0.001. More than 50% of participants indicated disgust for 5 of the 13 immoral scenarios, less than 50% of participants did so for all nine moral scenarios. The mean percentage of *yes* for *disgust* was 81% for the 13 scenarios With Pathogens; the corresponding figure for the nine scenarios Without Pathogens was 8%.

#### Relation between Immorality and Disgust

**FIGURE 1:**
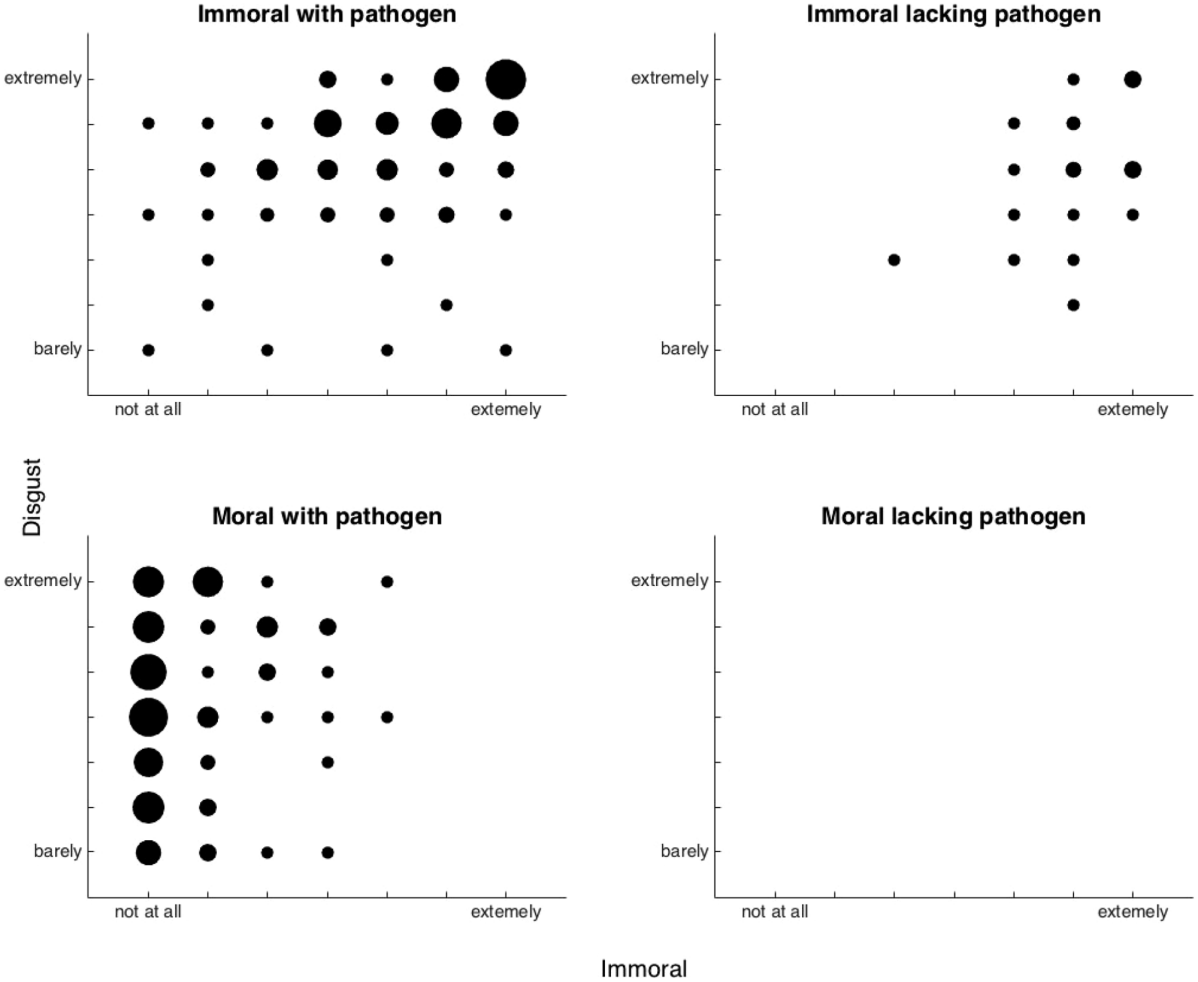
**Relation of immorality and disgust within each of the four groups of scenarios: Moral with Pathogens, Immoral with Pathogens, Moral without Pathogens, and Moral without Pathogens.** The size of the dot represents the number of cases; the larger the dot, the more cases.

**Figure 1** shows the relation of immorality and disgust within each of the four groups of scenarios: Moral with Pathogens, Immoral with Pathogens, Moral without Pathogens, and Moral without Pathogens. In testing our central hypotheses, one complication arose in that there was no variance in disgust scores for the Moral without Pathogen group of scenarios. Thus, in statistical analyses, we needed to circumvent this problem. A preliminary analysis thus examined whether disgust and immorality ratings varied separately with the three a priori groups of scenarios (i.e., omitting Moral without Pathogen group). There was a main effect of scenario group, *F*(2,267) = 627.88, a main effect of response (disgust versus immorality), *F*(2,267) = 1825.10, and most importantly, a scenario group by response interaction, *F*(2, 267) = 688.99, all *p*s < 0.001. Thus, immorality and disgust judgments differed significantly.

A correlational analysis examined the relation of immorality to disgust in more detail. Across the 22 scenarios, the correlation between mean intensity scores for disgust and those for immorality failed to reach significance, *r* = 0.08, n.s. (Similar results were obtained when dichotomous disgust and immorality scores were substituted; *r* = -0.18, n.s.) Thus, at the level of scenarios, immorality and disgust were unrelated.

At the level of individuals, a more mixed result was found. The first analysis used the intensity scores for both disgust and immorality. The correlation across scenarios (*N* = 22) was non-significant for 84 (out of 90) participants (range: -0.35 to 0.42); one significant correlation was negative, -0.44; the remaining five significant correlations ranged from 0.43–0.60. Thus, 5.5% of participants showed a significant (α = 0.05) positive correlation between disgust and immorality. A similar conclusion resulted when dichotomous ratings of disgust and immorality were substituted for quantitative ratings. Of the 90 participants, the phi correlation between disgust and immorality judgments (both scored as either 0 or 1) across scenarios (*N* = 22) was non-significant for 88 participants (range: -0.38 to 0.39); the two significant correlations were 0.45 and 0.51.

The next analysis examined whether those individuals who found a given scenario more immoral, relative to those who did not, were more likely to find it more disgusting. Of the 22 scenarios, four showed no variance in one or the other of the two judgments and were excluded from this analysis. For the remaining 18 scenarios, the correlation between disgust and immorality intensity judgments across subjects (*N* = 90) was non-significant for 15 scenarios (range *r*: -0.16 to 0.19), but significant for three scenarios—all from the Immoral with Pathogen group: scenario # 1, sex between young and old: *r* = 0.46, *p* < 0.001; scenario # 3, incest: *r* = 0.54, *p* < 0.001; and scenario # 4, brutal beating: *r* = 0.27, *p* < 0.02. For the remaining three scenarios in this group, the correlation was non-significant and for the group as a whole, the mean correlation was non-significant (mean *r* = 0.17, *p* = 0.12). For the remaining two a priori groups of scenarios, there was no relation between disgust and morality intensity judgments across subjects (*N* = 90): Immoral without Pathogens (mean *r* = 0.03, *p* = 0.79); Moral with Pathogens (mean *r* = -0.11, *p* = 0.31). In short, in 3 of the 18 scenarios examined, those individuals who found the scenario more immoral also found it more disgusting, but in 15 of the scenarios, no such relation was found. (Similar results were obtained when dichotomous ratings were substituted for quantitative ratings; correlations available upon request.)

Of particular interest is the relation between the dichotomous disgust scores and the intensity of immorality for the set of Immoral without Pathogen scenarios. This analysis addresses the question of whether those who found these scenarios disgusting found them more immoral. For the six scenarios, the correlation (*N* = 90) ranged from 0.05 to 0.14, with a mean of 0.09, all *p*s n.s.

#### Negative Reactions to Immoral Events

When not disgusted by immoral scenarios, the percentage of participants who felt another negative emotion ranged from 3 to 92 (mean = 48) for scenarios with pathogens and from 38 to 89 (mean = 68) for scenarios without pathogens; one felt happy for one of the 12 immoral scenarios. Immoral scenarios not found disgusting were nonetheless found negative whether they included or lacked pathogens, although the difference was significant (53% versus 74% of participants, respectively, *X*^2^ = 9.51, *p* < 0.01). Thus, participants disliked immoral scenarios.

Immoral scenarios not found disgusting were associated with the full range of negative emotions – including sadness and fear (emotions not generally linked to immorality but consistent with Study 1). For the Immoral scenarios with pathogens not found disgusting, the three most common emotions were fear, anger, and outrage (17, 13, and 13% of participants, respectively). For the Immoral scenarios without pathogens not found disgusting, the three were sadness, anger, and outrage (23, 22, and 21% of participants, respectively).

### Conclusion

Disgust was attributed largely to events — whether immoral or not — involving, either implicitly or explicitly, signs of potential exposure to pathogens. Disgust was rarely attributed to events — whether immoral or not — lacking potential exposure to pathogens. Specifically, immoral events without pathogens were rarely associated with disgust. Although disgust was not typically or strongly associated with immorality, we found some possible evidence of association, albeit weak. Disgust judgments correlated significantly with immorality judgments for 5.5% of participants (but with α = 0.05). Thus, there might exist a small subset of individuals who find more disgusting scenarios more immoral (or vice versa). We also found that there exists a subset of scenarios (3 of 18 = 16.67%) —two scenarios involving sex (incest, sex between a 17- and a 70-years-old), one violence (brutal beating of a boy) —in which judgments of disgust correlated with judgments of immorality.

**FIGURE 2:**
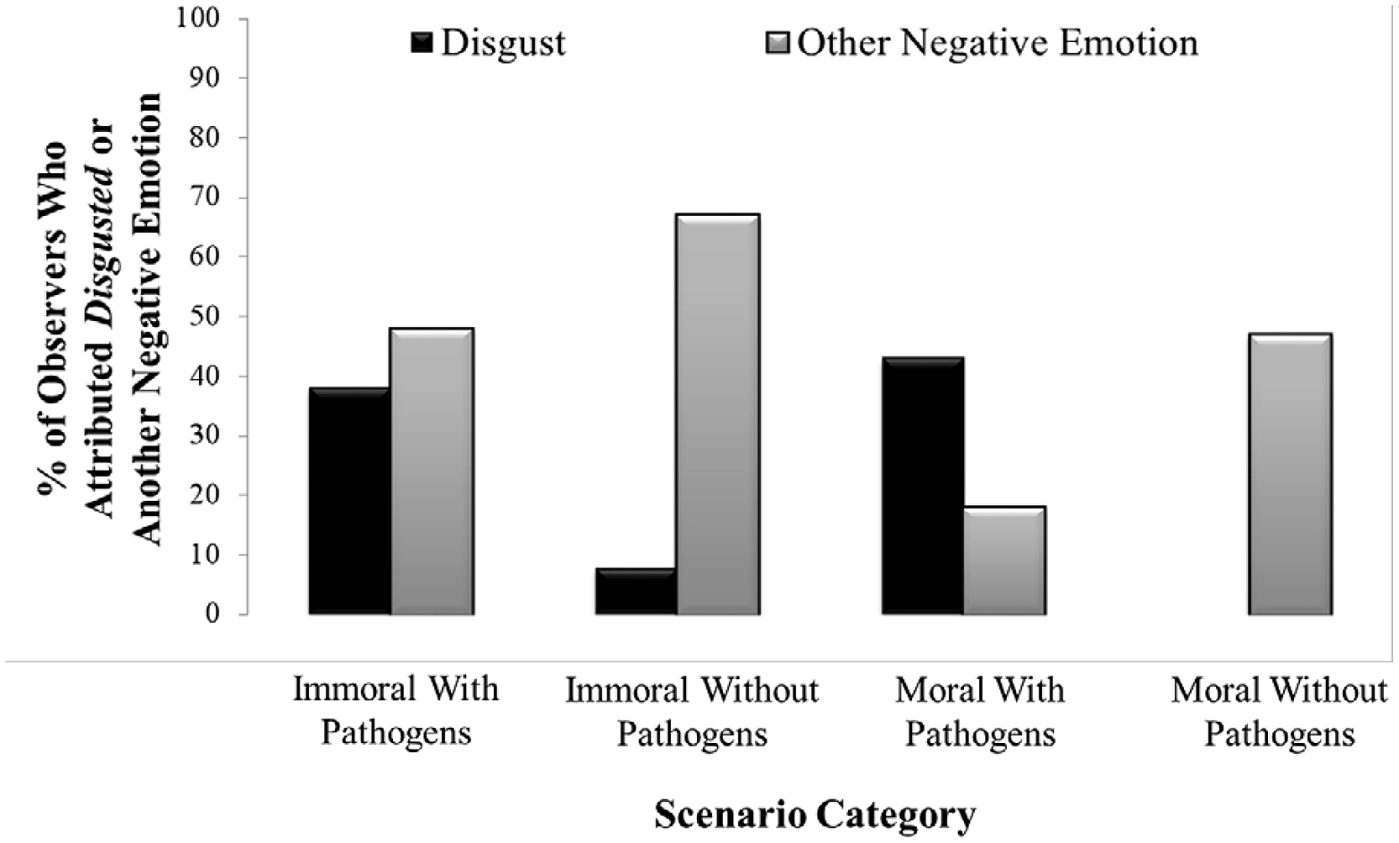
**Percentage of participants who selected disgusted or another negative emotion in each scenario category**.

Disgust was not the only negative emotion associated with judgments of immorality. **Figure 2** show that when participants did not associate disgust to an immoral scenario, they associated another negative emotion. And, indeed, anger and contempt were frequently used to describe reactions to various immoral events (Haidt, 2001, 2003; Nabi, 2002). Immoral events were associated with a full range of negative emotions, including not only disgust, anger, and contempt—emotions previously linked to the moral domain (Izard, 1977; Shweder et al., 1997; Rozin et al., 1999)—but also fear and sadness not previously linked to the moral domain. Indeed, all the negative emotions provided were chosen for the immoral scenarios. This range of negative emotions suggests that all these terms might be used to express a general feeling of dislike.

## General Discussion

In Study 1, all negative emotions were associated with judgments of immorality. The strongest association was with anger. With anger statistically controlled, disgust remained correlated with immorality, although modestly. Thus, the question arose: why is disgust associated with immorality? Study 2 found that disgust was attributed largely to events involving signs of potential exposure to pathogens, including morally good events (such as a nurse changing an elderly patient’s feces-covered sheets). When immorality and exposure to pathogens are separated, the association of disgust with immorality largely disappeared.

As were many studies in this research domain, our study was limited to a small set of verbally described scenarios and verbal ratings. Perhaps behavioral or physiological measures in actual situations (Chapman et al., 2009) might uncover different results. Nevertheless, our small correlational study does raise questions about an important set of ideas about morality and disgust.

Our studies showed that although disgust was not typically or strongly associated with immorality, some association, albeit weak, does exist. In Study 2, for example, disgust judgments correlated significantly with immorality judgments for 5.5% of participants (but with α = 0.05). Thus, there might exist a small subset of individuals who find more disgusting scenarios more immoral. The question is whether this association is a matter of sampling error or, if not, how to interpret that association in light of the general overall lack of association. One possibility is an individual difference variable in attentiveness to internal physical states. Schnall et al. (2008, Study 2) offered evidence of individual differences in the influence of disgust reactions on subsequent moral judgments. Specifically, only those individuals who scored high on general attentiveness to their internal physical states judged moral violations more harshly when seated at a dirty desk than a clean desk. Such evidence, however, does not necessarily implicate disgust specifically because sitting at a dirty desk is also simply unpleasant.

Study 2 also suggested that there exists a subset of scenarios (3 of 18 = 16.67%) within which judgments of disgust correlated with judgments of immorality. Again, the question is whether this association is a matter of sampling error or, if not, how to interpret that association in light of the general overall lack of association. One possibility is in situations with pathogens those individuals more sensitive to the presence of pathogens found the situation more disgusting and the presence of pathogens more harmful and therefore more immoral. Another possibility is that immoral events are more disliked than are moral events and that some individuals express their dislike with the word *disgust*. This possibility is consistent with the observation that the word *disgust* is often used metaphorically (Royzman and Sabini, 2001; Bloom, 2004). Indeed, Zhong and Liljenquist (2006, p. 1451) cited Lakoff’s (1987) work on metaphor to speculate that “individuals are predisposed to use categories based on bodily experience (such as clean versus dirty) to construct complex social categories (such as moral versus immoral).”

Another possible relation between disgust and immorality is that disgust, like any negative feeling, leads to more negative judgments in general (e.g., Forgas et al., 1984; Baron, 1987). This possibility is consistent with the finding that experimentally inducing disgust (with fart spray, for example) leads people to judge others’ moral violations more harshly (Eskine et al., 2011; Rottman and Kelemen, 2012). Thus, in these studies, the disgust-immorality association is an example of the well-established general principle that negative affect leads to negative judgments. In an attempt to rule out this more general interpretation, two studies included a sad control group (Horberg et al., 2009, Study 2; Schnall et al., 2008, Study 4). These studies found harsher moral judgments in the disgust condition than in the sad condition, but several concerns remain: for one, disgust might have been induced with more intensity than was sadness. In Schnall et al. (2008), for example, the mean intensity of self-reported disgust for the disgust condition (on a scale from 0 to 21) was 11.25, whereas that for sadness in the sadness condition was 4.72 (but see Horberg et al., 2009). Another concern is that sadness includes lower arousal than does disgust, which might lessen its impact on subsequent judgments.

Whether or not there is some association between disgust and judgments of immorality, the association found here was rare and, at most, weak. In both studies, we found disgust in scenarios that were not found immoral and immoral scenarios that were not disgusting. Thus, what is immoral is not necessarily disgusting, and what is disgusting is not necessarily immoral. Perhaps no theorist meant to claim otherwise, but these findings raise a question for those who theorize that disgust is involved in the judgment of morality (Wheatley and Haidt, 2005; Zhong and Liljenquist, 2006; Schnall et al., 2008; Chapman et al., 2009; Herz and Hinds, 2013): what in addition to disgust determines whether the event is found moral or immoral? Because, at most, only some disgusting events are found immoral, what differentiates the cases in which disgust is associated with immorality from those in which disgust is not? Similarly, at most, only some immoral events were found disgusting. What differentiates the cases in which an immoral event is found disgusting from the cases in which the immoral event is not?

Disgust was not the only negative emotion associated with judgments of immorality. And, indeed, *anger* and *contempt* are frequently used to describe reactions to various immoral events (Haidt, 2001, 2003; Nabi, 2002). Still, we found that immoral events were associated with a full range of negative emotions, including not only disgust, anger, and contempt – emotions previously linked to the moral domain (Izard, 1977; Shweder et al., 1997; Rozin et al., 1999; Russell and Giner-Sorolla, 2013) – but also fear and sadness not previously linked to the moral domain. Indeed, all the negative emotions provided were chosen for the immoral scenarios. This range of negative emotions suggests that all these terms were used to express a general feeling of dislike, consistent with the notion that the emotion of disgust has vaguely defined boundaries and conveys disapproval across many disparate domains (Strohminger, 2014). If so, then the use of *disgust* too might express a general dislike.

Immoral events were associated with a range of negative emotions in both studies, but we speculate that these same negative emotions occur as reactions to events not deemed immoral. The association between negative emotions and morality judgments could be tested with the simple correlational design used here. If our speculation is supported, the question then arises: what in addition to a negative emotion determines whether the event is found moral or immoral? Some theorists have suggested that disgust is the basis of judgment of immorality in only certain domains. Our interpretation that immoral scenarios are judged disgusting when they involve signs of potential pathogens overlaps with hypotheses that immoral scenarios are judged immoral when they involve purity violations – a more psychological form of contamination defined as spiritual defilement or disrespecting the “natural order of things” and the sanctity of one’s body (Rozin et al., 1999, p. 576). Despite this overlap, our hypothesis is fundamentally different. Studies that specifically linked disgust to purity violations often used as examples various taboos that involve sexual- or food-related behaviors (e.g., Haidt et al., 1994, 1997; Rozin et al., 1999; Gutierrez and Giner-Sorolla, 2007; Olatunji et al., 2007; Horberg et al., 2009). Such violations of “purity” involve potential pathogens (or vectors or consequences of pathogens). On the account we favor, the potential pathogens are the simplest explanation, and there is no need to invoke “purity” in these cases. Royzman et al. (2014) recently offered empirical evidence supporting just such an interpretation. In future research, pathogens and purity must be disentangled by examining disgust attributions to immoral events that involve purity violations, but lack pathogens or their correlates. Such separation may be more difficult than it seems, however, because disgust is not elicited directly by disease and toxins, but by signs of them. Thus, verbal assurances that the elicitor is sterilized or otherwise protected from disease may leave intact the evolutionarily based signs of disease. A further difficulty is that the word *disgust* is used broadly to express anger and dislike (Nabi, 2002).

An association between disgust and immorality has been bolstered by an evolutionary argument. The basic idea is that the disgust mechanism for rejecting potentially harmful foods was co-opted to respond to potentially harmful social interactions (Tybur et al., 2009). To our knowledge, the theory of re-purposing of the disgust mechanism was supported by no direct evidence, but indirectly by finding a purportedly primal disgust reaction (e.g., facial expression) purportedly elicited by moral actions (Cannon et al., 2011; Chapman and Anderson, 2013; but see Pochedly et al., in preparation), and also by research showing that participants tasting bitter substances make harsher moral evaluations (Eskine et al., 2011). The current studies, however, challenge the purportedly evolved association between immoral actions and disgust specifically (above and beyond anger). If the association of immorality with disgust is as weak and limited as suggested by the current studies, an evolutionary explanation for that association is neither necessary nor useful.

The association between disgust and morality is weak. It is sufficiently weak that a direct link is questionable. What link exists would then be indirect. One indirect link would be simply coincidence: events found immoral sometimes include signs — blood, saliva, semen — of possible pathogens. Another indirect link is the semantics of the word *disgust*, which is used to convey a range of feelings including anger and disapproval (Nabi, 2002). It remains to be seen if there is any other link.

## Conflict of Interest Statement

The authors declare that the research was conducted in the absence of any commercial or financial relationships that could be construed as a potential conflict of interest.
