# Supplementary material for: On the limits of the relation of disgust to judgments of immorality
Source: Front Psychol. 2015 Jul 15;6:951. doi: 10.3389/fpsyg.2015.00951 (PMC4502534; doi:10.3389/fpsyg.2015.00951)
Supplement: Supplementary file 1 [file Table_1.PDF]

Appendix A

	Emotion Judgment (mean intensity)				
	Morally Bad Stories				
	Disgust	Anger	Sadness	Fear	Happiness
**Casey,** a 25-year-old, offers sexual favors to her married attractive boss to get a promotion.	4.3	3.3	1.8	0.2	0
**Casey** is a nurse in an under-funded charity clinic. She is responsible for giving patients injections. One day, she finds that the clinic has run out of new needles. She opens the disposal container of dirty needles to use.	4.9	4.4	3.2	4.2	0.4
**Casey** is, a chef in a small restaurant. He spits in the food of customers he does not like.	5.6	4.2	1.3	2.1	0
**Casey** is a food server at a local restaurant. He shows up for work one busy night even though he has a runny nose and a bad cough because he expects large tips. He often ends up sneezing on the plates while carrying them out to customers.	4.9	3.9	0.9	2.2	0.5
**Casey**, who works for a large company, dumps garbage into a drinking water	5.8	5.1	2.6	2.3	0

reservoir rather than take it to the disposal center					
**Casey**, your manager, gave his friends in the company large raises. You got no raise.	1.7	2.6	4.2	0	0
**Casey**, your manager, gave you much misleading information about products the company sells. As a result, you inadvertently mislead customers into purchasing highly overpriced products. Your reputation is badly damaged and some of your best customers refuse to do business with you ever again because of your manager's instructions.	2.0	1.8	2.8	0	0
You voted for **Casey**, your congressman. You later find out that **Casey** took a large campaign contribution from a company that competes with yours and then voted for their agenda. Your company goes out of business.	1.0	2.3	3.7	1.0	0.5
**Casey** is a perpetually bad driver. One night, **Casey** was texting on his cell phone and crashed into your parked car. He drove off and was never caught.	2.1	2.0	3.4	1.7	0.6
**Casey** is a fraudulent investor. He tricks you into investing your money in stocks that are worthless. You lose a lot of money.	3.6	4.0	4.4	1.8	0.1

**Casey**, an overprotective father did not tell his daughter that her much loved grandmother was on her deathbed. **Casey** also ignored the grandmother's request to see her grandchild because he did not want to upset his daughter. The grandmother died and **Casey**'s daughter never got to say good-bye.	4.7	5.3	3.4	0.2	0
**Casey** couldn't bring himself to forgive – or even speak to-- his dying father who was seldom around because of his demanding career as a world class brain surgeon.	4.0	5.4	3.2	2.8	0.1
**Casey** has been unexpectedly laid off. He sells family heirlooms that everyone in the family treasures in order to pay the mortgage.	4.4	5.2	4.8	2.9	0.1
**Casey** grew up in a troubled home with an alcoholic and abusive father. Casey is emotionally cold and detached from his own wife and children.	4.3	5.6	3.0	1.0	0
Casey, a teen who was severely bullied his whole life, snapped and becomes involved in a school shooting in which he also kills himself.	5.3	5.6	3.6	2.3	0.8
You live next door to **Casey** and his daughter. **Casey** has a history of family violence. Recently, you've seen **Casey** carrying a gun. Now you	4.0	3.8	4.0	5.3	0.7

hear **Casey** yelling at his daughter.					
**Casey**, your cousin, offers you a lot of money. He wants you to deliver a large quantity of cocaine to an address in another part of town. You have never been involved in anything like this before and are hesitant, but do it anyway. You are driving in your car when you suddenly find yourself surrounded by members of a rival gang with guns drawn.	2.7	3.7	1.5	5.4	0.5
**Casey** has all his life lit fires and has been responsible for serious ones. You have a friend who lives near **Casey** and goes to **Casey**'s school.	1.2	2.5	2.4	4.4	0.9
**Casey** is your friend. He, offers you a ride after a party. You get in the car, but soon realize that he is drunk. He starts to swerve on a street where lots of kids are playing.	3.9	4.4	2.5	5.5	0.2
**Casey**, your friend, has been gambling heavily and owes the gang money he can't pay.	3.1	2.7	4.4	5.1	0.5
Morally Good Stories					
**Casey**, a good friend of yours, is a physician. One day in the hospital, she finds that her patient has had diarrhea. The staff are all busy, and	3.5	0.3	0.6	0.8	0

so **Casey** quickly changes the patient's clothes and bed sheets. **Casey** gets feces on her bare hands and arms.					
**Casey** is babysitting a sick infant. **Casey** tries to calm the crying baby when it pukes down her neck and back.	2.4	0.4	1.3	0.4	0.4
You and **Casey** are walking along the sidewalk when an obviously drunken, middle-aged man walks toward you. The man then stumbles into Casey and accidentally knocks them both to the ground. With great difficulty, Casey helps him up, but now smells like the drunken man's urine, vomit, and feces.	3.7	1.3	1.8	0.6	0
**Casey** moves an injured skunk off the road so that it does not get hit by a car. **Casey** gets blood and puss all over his hands and shirt, and he gets sprayed by the skunk.	3.8	0.7	2.3	0.9	0.9
**Casey** arrives at a friend's house, who has just eaten leftovers for dinner. Casey's friend starts to feel nauseous and dizzy. **Casey** walks over to help his friend and slips in a pool of vomit.	4.3	0.7	1.8	1.4	0.2
You are traveling with a co-worker to an important business meeting in which you are competing with other companies for a contract. You go pick	0.1	1.1	4.6	2.7	0

up your co-worker to go to the airport, but your co-worker is not ready, causing you to get to the airport later than planned. That day, the country was put on a high alert, and **Casey**, the head of TSA for the airport, ordered that all passengers be subjected to a search and background check. All the agents are extremely thorough and methodical. The agent checking you works very slowly, gets sidetracked by other tasks, and takes hours to finish the search. You end up missing the meeting and losing the contract.					
You get to the airport just in time to check in for a flight. **Casey**, the airline agent explains that all flights were overbooked and your seat was given to a person rushing home to see his elderly mother. It was the last flight for the night and you have to spend the night at the airport. You are tired and stressed out.	0	0.6	4.2	0.7	0.7
You are driving to your best friend's wedding when you witness a terrible car accident. **Casey**, a police officer, orders you to the police station to make an official witness statement, and you miss the wedding.	0.3	0.1	3.8	0.9	0
You have been unemployed for months and you are on your way to an	0.7	1.4	4.1	0.8	0.6

important job interview. **Casey**, the mayor, orders construction workers to fix power lines to a large hospital that have been damaged due to a storm. This causes traffic delays that make you miss the interview and you do not get the job.					
**Casey**, your neighbor, takes in stray, injured animals that would otherwise be euthanized and finds families to adopt them. The animals constantly make noise and keep you up all night.	0	0.7	3.1	0.5	0.6
**Casey** spent days and nights in a hospital taking care of her sick niece, who was slowly dying of cancer.	2.7	3.9	1.5	0.7	0
**Casey**, a mother, put her baby up for adoption because her own medical condition precluded her from caring for the baby.	1.7	4.1	2.7	0.1	0
**Casey**, a volunteer, was re-building homes destroyed by a hurricane. He was badly injured in a construction accident.	1.9	3.4	3.6	0.5	0.6
**Casey**, a loving mother, is asked by her son to provide a false story as alibi for him for a crime that he admitted he did. She considers it, but can’t bring herself to lie because she feels lying is wrong. She knows her son will go to	2.0	4.1	2.2	0.1	0

prison.					
**Casey**, a medical researcher, had tried to find a cure for a debilitating disease, but contracted the disease himself**,** and cannot return to his work for months.	0.5	3.0	1.2	0.6	2.3
**Casey**, your friend, is a young firefighter. **Casey** prepares to go into a large burning house to save those inside.	0	0.2	1.5	4.4	0.2
**Casey**, your friend, is a dedicated volunteer at an HIV clinic. One day, **Casey** rushes to help a badly injured patient without having time to put on gloves, risking contracting HIV.	1.8	2.8	2.0	3.9	0.9
**Casey**, your friend, a young doctor, is doing charity work in a foreign country when an unexpected war breaks out.	2.5	3.9	3.8	5.2	0
**Casey**, your friend, jumps into a river with a strong undercurrent to save her fallen child as you watch.	0	0.3	2.1	4.8	0.7
**Casey**, your best friend, is doing charity work in a high-conflict area. You hear about a drive-by shooting spree in the area, but you have no way to contact **Casey**.	2.1	2.3	2.3	5.4	0
