# Supplementary material for: On the limits of the relation of disgust to judgments of immorality
Source: Front Psychol. 2015 Jul 15;6:951. doi: 10.3389/fpsyg.2015.00951 (PMC4502534; doi:10.3389/fpsyg.2015.00951)
Supplement: Supplementary file 2 [file Table_2.PDF]

Appendix B

Emotion-Eliciting Scenarios and Their Mean Immorality and Disgust Ratings (Study 2)

	Immorality		Disgust	
Scenarios	% Yes	Mean intensity	% Yes	Mean intensity
Immoral With Pathogen				
1. You hear about a **17-year-old girl** who is having an intimate relationship with a 70-year-old man.	99%	3.9	81%	4.5
2. You witness as **two teens** mutilate a dead puppy with their bare hands for fun.	100%	5.6	41%	2.6
3. You hear about a **person** who is involved in an incestuous relationship.	94%	4.7	90%	5.1
4. You witness a 12-year-old boy pull out a knife against an **older man** who then brutally beats up the young boy.	97%	3.4	7%	0.4
5. You are present during a bank robbery in which the **robber** beats up the bank teller.	99%	5.4	2%	0.1
6. You are in a car that gets cut off. **Your driver** pursues the other car and intentionally causes the other driver to get into an accident.	100%	4.9	9%	0.5
Immoral Lacking Pathogen				

7. You hear about a **homeless woman** stealing a stranger's wallet so that she can feed her two young children.	99%	3.1	1%	0.0
8. You hear about a poor **immigrant student** who lies to her academic institution about her citizenship status in order to get government-funded financial aid.	99%	3.1	4%	0.2
9. You hear about a **woman** whose husband emotionally abuses her. She frames her husband for a crime he didn't commit; as a result, he is sentenced to ten years in prison.	100%	3.8	4%	0.2
10. You hear about a wealthy **business owner** who purposefully opens his new big restaurant next to an old small family-owned restaurant to maximize his business.	98%	4.4	8%	0.4
11. You hear about a **cancer researcher** who makes up data to support his theory to increase sales of his pharmaceutical product.	100%	5.1	16%	0.8
12. You hear about a corporate **CEO** who embezzles money from the company bank account to take luxurious vacations; as a result, many employees lose their jobs.	100%	5.5	12%	0.6
Moral With Pathogen				

13. You witness **someone** carrying an injured kitten off the road.	3%	0.1	1%	0.1
14. You witness a **nurse** removing sheets that an elderly woman has defecated all over; the nurse accidentally gets some feces on herself.	6%	0.1	63%	2.6
15. You watch your **newly married friends** French-kiss at the altar.	34%	0.5	8%	0.2
16. You watch as a **mother** cleans up after her sick baby who just threw-up all over her.	1%	0.0	38%	0.8
17. You watch as a **woman** uses CPR in an effort to save a man who is not breathing.	0%	0.0	0%	0.0
18. You witness a **stranger** on the bus eating his boogers.	47%	0.7	88%	3.9
19. You watch your **friend** takes a bite into an apple and find a slimy worm inside.	3%	0.1	86%	3.9
Moral Lacking Pathogen				
20. You hear about a **teacher** who works extra hours for free to tutor inner city kids.	0%	0.0	0%	0.0
21. You watch as a young **firefighter** prepares to go into a large burning house to save those inside.	2%	0.0	0%	0.0
22. You are present in court when a mother	43%	0.8	0%	0.00

hears the **judge** sentence her guilty son to 15 years in prison.
